# MicroRNAs play an essential role in autophagy regulation in various disease phenotypes

**DOI:** 10.1002/biof.1555

**Published:** 2019-08-16

**Authors:** Yunyi Zhao, Ze Wang, Wenhui Zhang, Linbo Zhang

**Affiliations:** ^1^ Laboratory of Pathogenic Microbiology and Immunology College of Life Science, Jilin Agricultural University Changchun China; ^2^ Ministry of Education, Engineering Research Center for Bioreactor and Pharmaceutical Development Jilin Agricultural University Changchun China

**Keywords:** autophagy, disease, microRNAs, regulation

## Abstract

Autophagy is a highly conserved catabolic process and fundamental biological process in eukaryotic cells. It recycles intracellular components to provide nutrients during starvation and maintains quality control of organelles and proteins. In addition, autophagy is a well‐organized homeostatic cellular process that is responsible for the removal of damaged organelles and intracellular pathogens. Moreover, it also modulates the innate and adaptive immune systems. Micro ribonucleic acids (microRNAs) are a mature class of post‐transcriptional modulators that are widely expressed in tissues and organs. And, it can suppress gene expression by targeting messenger RNAs for translational repression or, at a lesser extent, degradation. Research indicates that microRNAs regulate autophagy through different pathways, playing an essential role in the treatment of various diseases. It is an important regulator of fundamental cellular processes such as proliferation, autophagy, and cell apoptosis. In this review article, we first review the current knowledge of autophagy and the function of microRNAs. Then, we summarize the mechanism of autophagy and the signaling pathways related to autophagy by citing at least the main proteins involved in the different phases of the process. Second, we introduce other members of RNA and report some examples in various pathologies. Finally, we review the current literature regarding microRNA‐based therapies for cancer, atherosclerosis, cardiac disease, tuberculosis, and viral diseases. MicroRNAs can cause autophagy upregulation or downregulation by targeting genes or affecting autophagy‐related signaling pathways. Therefore, the microRNAs have a huge potential in autophagy regulation, and it is the function as diagnostic and prognostic markers.

**Abbreviations**
Ago: Argonaute
Akt: protein kinase B
ALKBH5: AlkB homolog 5
AMPK: AMP‐activated protein kinase
AS: atherosclerosis
ATG: autophagy‐related gene
Bcl‐2: B cell lymphoma 2
BVDV: bovine viral diarrhea virus
CAB39: calcium‐binding protein 39
CACNA2D3: alpha‐2/delta subunit 3
CH: cardiac hypertrophy
circRNAs: circular RNAs
CtsS: cathepsin S
DENV: dengue virus
DRAM2: DNA damage‐regulated autophagy modulator 2
EV71: Enterovirus 71
FIP200: FAK family‐interacting protein of 200 kDa
GRSF1: G‐rich RNA sequence binding factor 1
HAECs: human aortic endothelial cells
HBV: hepatitis B virus
HCM: hypertrophic cardiomyopathy
HCV: hepatitis C virus
HFD: high‐fat diet
HUVECs: human umbilical vein endothelial cells
IFN‐γ: type III interferon γ
IGF‐1R: insulin‐like growth factor 1 receptor
JNK: c‐Jun N‐terminal kinase
LC3: microtubule‐associated protein 1A/1B‐light chain 3
LMNB1: lamin B1
LN: Lupus nephritis
lncRNAs: long noncoding RNAs
MDBK: Madin‐Darby bovine kidney
MDM2: mouse double minute 2
microRNAs: micro ribonucleic acids
MTB: *Mycobacterium tuberculosis*
mTORC1: mTOR complex 1
m6A: N6‐methyladenosine
Notch 1: Notch receptor 1
NSCLC: non‐small cell lung cancer
nt: nucleotides
OS: osteosarcoma
ox‐LDL: oxidized low‐density lipoprotein
PCVAD: porcine circovirus‐associated disease
PCV2: porcine circovirus type 2
PDAC: pancreas ductal adenocarcinoma
PE: phosphatidylethanolamine
PI3K: phosphoinositide 3‐kinase
PI3P: phosphatidylinositol‐3‐phosphate
pri‐miRNAs: primary miRNA transcripts
RV: rotavirus
SAPK: stress‐activated protein kinase
SKP2: S‐phase kinase‐associated protein 2
SNHG5: snoRNA host gene 5
SNHG14: small nucleolar RNA host gene 14
SPHK1: Sphingosine kinase 1
TB: tuberculosis
TGF‐β: transforming growth factor
TLRs: Toll‐like receptors
TNA: threose nucleic acid
TRAF6: tumor necrosis factor receptor‐associated factor 6
TRPV3: transient receptor potential vanniloid 3
TSC1: tuberous sclerosis complex 1
TSC2: tuberous sclerosis complex 2
ULK1: Unc51‐like kinase 1
3′‐UTR: 3′‐untranslated region
VEGFA: vascular endothelial growth factor A
XIAP: X‐linked inhibitor of apoptosis

## 1 INTRODUCTION

Autophagy is a well‐organized homeostatic cellular process through which diverse cytoplasmic cargos are captured and destroyed to replenish energy sources and amino acids during metabolic stress.^1^, ^2^ Autophagy process comprises three steps: the first step is when autophagy‐related gene (ATG) drives the cup‐shaped isolation membrane to decompose into phagophores with omegasome, an autophagic intermediate formed by the endoplasmic reticulum, in the cytosol. Next, the phagophores sequester the cell contents used for autophagy and form autophagosome with a double‐membrane structure by elongation and closure. Finally, autophagosomes and lysosomes keep approaching to each other. Under the mediated by soluble *N*‐ethylmaleimide sensitive factor attachment protein receptor, the external autophagosome membrane is fused with a lysosome. The inner autophagosome membrane is degraded by lysosome to complete fusion, autophagolysosome is formed, and the contents of autophagosomes are exposed to the lysosome. Then, cargo is degraded by autophagosome acidification and lysosomal hydrolase.^3^, ^4^, ^5^, ^6^, ^7^

Autophagy is a tightly regulated multi‐step process that is regulated by several signaling cascades. (A): Being the critical regulator of autophagy initiation, MTORC1 (mTOR [mechanistic target of rapamycin], mTORC1 [mTOR complex 1]) negatively phosphorylates the threonine/serine kinase ULK1 (Unc51‐like kinase 1) complex and ATG13, two members of the ULK1 complex that also includes ATG101 and FIP200 (FAK family‐interacting protein of 200 kDa). Following cellular stress, MTORC1 is inactivated, resulting in the release of the ULK1 complex, its dephosphorylation, and subsequent activation of ULK1 kinase activity. ULK1 then phosphorylates itself and its partners, FIP200 and ATG13, leading to the activation of autophagy by the formation of the ULK‐ATG13‐ATG101‐FIP200 complex.^8^, ^9^, ^10^, ^11^ (B): Along with the phosphoinositide 3‐kinase regulatory subunit 4, ATG14, and the scaffold protein Bcl2‐interacting protein 1, phosphatidylinositol 3‐kinase catalytic subunit type 3 forms the class III PI3K complex, which generates a membrane domain enriched in PtsIns3P and creates PI3P (phosphatidylinositol‐3‐phosphate) at the site of nucleation of the phagophore which leads to the binding of PI3P binding proteins, and the subsequent recruitment of proteins involved in “the ubiquitin‐like protein conjugation systems” to the isolation membrane.^12^, ^13^, ^14^ (C): ATG5 is a lysine residue forms a covalent conjugation with a C‐terminal glycine residue of ATG12 is catalyzed by E1‐like enzyme Atg7 and E2 like enzyme Atg10, forming an autophagosomal precursor. Typically, the ATG5‐ATG12‐ATG16L1 complex forms the autophagosome membrane in two ways: by directly binding with the membrane or by involving in the LC3‐PE conjugation pathway. The ATG5‐ATG12 conjugate may act as an E3‐like enzyme for the conjugation of microtubule‐associated protein 1A/1B‐light chain 3 (LC3) to phosphatidylethanolamine (PE) and, together with ATG16L1, accomplishes the deposition of LC3‐PE on the phagosome. Before conjugation, LC3 is processed by ATG4B cysteine peptidase. ATG4B is responsible for cleavage of the carboxy terminus of the newly synthesized pro‐LC3 to provide its cytosolic form of LC3 (LC3‐I). Then, LC3‐I is also activated by Atg7, transferred to Atg3, and modified to an autophagosomal membrane‐bound form of LC3 (LC3‐II). Elongation of the phagophore is aided byLC3 II, ATG9, the ATG12–ATG5‐ATG16L1 complex, and the class III PtdIns3K complex. Eventually, the expanding membrane closes around its cargo to form an autophagosome, and LC3‐II is cleaved from the outer membrane of this structure.^7^, ^15^, ^16^, ^17^

AMP‐activated protein kinase (AMPK) is a central regulator of the cellular response to low energy levels. AMPK negatively regulates mTORC1 activity by two complementary actions. First, AMPK activates TSC2 (tuberous sclerosis complex 2) by phosphorylating Ser1345 and Thr1227 residues, thus reducing Ras homologue enriched in brain activity and facilitating the assembly of TSC1/TSC2 heterodimer, which negatively affects mTORC1 activity. Another AMPK can inhibit mTORC1 by direct phosphorylation of Ser722 and Ser792 residues, thus activating autophagy indirectly. Indeed, AMPK increases ULK1 activity to regulate autophagy by direct phosphorylation of Ser637, Ser555, Ser467, and Thr574 positively, which increases the recruitment of ATG to the membrane domains in which autophagosome formation occurs.^13^, ^18^, ^19^, ^20^ mTORC1 senses and responds to fluctuations in intracellular and extracellular nutrient levels, primarily amino acids as well as oxygen levels, growth factor signaling, and cellular energy. In the absence of activating stimulation, autophagy is induced by dissociation of mTORC1 complex from the ULK1 complex, thereby relieving the inhibition of ULK1, which is then responsible for its phosphorylation and phosphorylation of FIP200, Atg13, and Raptor. ULK1 is then able to activate this PI3K complex and promote autophagosome synthesis.^21^ Upon activation, mTORC1 promotes anabolic processes through phosphorylation of its eukaryotic translation initiation factor 4E binding protein and downstream effectors ribosomal protein S6 kinase, thereby inducing cell proliferation and growth.^20^, ^22^ When growth factors/amino acids or energy are abundant, mTORC1 represses autophagic process through inhibitory phosphorylation of ATG13, which reduces the activity of a mTORC1 direct target, ULK1, thereby decreasing the rate of autophagosome formation.^21^, ^23^

Also, autophagy is not only a way for cells to gain nutrients by degrading cellular components during starvation but is also an important defense mechanism against intracellular pathogens. During host cell invasion, the intracellular pathogen is initially absorbed into a membrane surrounded compartment. Upon detection of microorganisms inside the undamaged vacuole by Toll‐like receptors (TLRs), LC3/GABARAP can become directly conjugated to the limiting membrane of the bacterium‐containing vacuole in a process termed LC3‐assisted phagocytosis. Conjugation of LC3/GABARAP to pathogen‐containing vacuoles promotes content degradation by enhancing lysosomal fusion.^24^, ^25^, ^26^, ^27^ Autophagy assists in the secretion of antimicrobial peptides and proteins and intracellular viral restriction factors. For instance, several reports revealed that some populations of tuberculosis patients display polymorphisms in genes linked to the autophagy pathway, namely, the human immunity‐related GTPase M, type III interferon γ (IFN‐γ), IFN‐γ receptor, endosomal TLR8, Vitamin D3, and ATP receptor P2X7R, suggesting that some individuals might be more prone to develop tuberculosis due to a defective autophagic response. Finally, this process can also be used for the activation of the adaptive immune response via the fusion of autophagosomes with antigen‐loading compartments for major histocompatibility complex class II molecules.^28^, ^29^, ^30^

Non‐encoding RNAs that are not translated into proteins can be divided into linear, for example, long noncoding RNAs (lncRNAs) and microRNAs (miRNAs), or circular, for example, circular RNAs (circRNAs) are based on the linearity of transcripts. LncRNAs are generally longer than 200 nucleotides (nt), are located in the cytoplasm or nucleus, make up the largest proportion of mammalian noncoding transcriptome, and have important functions in cellular processes such as motility, cell proliferation, and apoptosis. LncRNAs are differentially expressed in various tissues and are able to associate with proteins to modulate their functions, modulate transcription of protein‐coding genes, and control protein synthesis and RNA transport and maturation. Although they are polyadenylated, capped, spliced, and transcribed, lncRNAs do not translate into proteins. Most lncRNAs in nuclear organization regulate gene transcription through recruiting proteins which modify the chromatin and thus trigger chromatin conformation changes.^31^, ^32^, ^33^, ^34^, ^35^ H19 is a 2.3‐kb lncRNA which is transcribed from insulin‐like growth factor‐II genomic imprinted cluster located on human chromosome 11p15.5; its expression is high in embryonic organs and greatly reduced or absent in most adult tissues. Overexpression of H19 in myocardial tissues caused decreases in inflammation, apoptosis, autophagy, and oxidative stress, leading to the amelioration of diabetic cardiomyopathy.^36^ MEG3 is believed to be a tumor‐suppressive lncRNA and correlated negatively with poor prognosis in lung cancer. In vitro experiment showed that upregulation of MEG3 increased the sensitivity of A549 cells to cisplatin by activation of the WNT/β‐catenin signaling pathway, whereas downregulation of MEG3 caused the opposite effect.^37^ When lncRNAs in plasma from myocardial infarction patients were profiled, a mitochondrial lncRNA named long intergenic noncoding RNA predicting cardiac remodeling was found to correlate with mortality caused by cardiovascular diseases significantly and could thus be considered as a biomarker.^38^ lncRNA (CARL) suppressed mitochondrial fission and apoptosis in vitro and reduced reperfusion injury/ischemia in vivo. Mechanistically, Wang et al. showed that CARL binds to miR‐539 and acts as a sponge to prevent anoxia‐induced mitochondrial fission. lncRNA (AK048451) induced apoptosis in vivo and cardiomyocyte hypertrophy in vitro. The mechanism of action was attributed to binding of miR‐489, which derepresses the miR‐489 target Myeloid differentiation primary response gene 88 to regulate cardiomyocyte hypertrophy.^39^

circRNAs are produced through non‐canonical alternative splicing and form covalently closed RNA loops that the 3′ and 5′ ends are joined together. The length of spliced circRNA ranges from under 100 nt to over 4,000 nt. Most of the circRNAs are generated from exons of protein‐coding genes and are single‐stranded transcripts that are ubiquitously expressed in all eukaryotes and even prokaryotic archaea. Generally speaking, circRNAs are very stable. They have been identified as splicing activities or transcriptional regulators, scaffolds of proteins, miRNA sponges, and protect mRNA targets from cleavage and translation repression induced by miRNAs. Moreover, many circRNAs are tissue‐specifically expressed, highly conserved, and can be detected in many kinds of body fluids.^40^, ^41^, ^42^, ^43^, ^44^ circRNA_100782 was markedly upregulated in pancreatic ductal adenocarcinoma tissue and regulated BxPC3 pancreatic cancer cells proliferation by acting as miR‐124 sponge through the IL6–STAT3 pathway.^45^ Functional experiments revealed circRNA_010567 silencing could upregulate miR‐141 and downregulate TGF‐β1 expression, play an important regulatory role in the diabetic mice myocardial fibrosis model, and suppress fibrosis‐associated protein resection in cardiac fibroblasts.^46^ The systemic lupus erythematosus (SLE) is the prototypic human autoimmune disease characterized by the presence of autoantibodies against several self‐antigens, which causes serious injury to various organs or systems. Lupus nephritis (LN) is a major risk factor for overall mortality and morbidity in SLE, affecting up to 70% of SLE patients, and about 10–30% of LN will progress to end‐stage renal failure. The upregulated plasma circRNA_002453 level in patients with LN is associated with the severity of renal involvement and may also serve as a potential biomarker for the diagnosis of patients with LN.^47^ circRNAs‐MSR regulated tumor necrosis factor‐alpha expression and participated in the chondrocyte extracellular matrix degradation process. Liu et al. propose that the inhibition of circRNAs‐MSR could inhibit the degradation of chondrocyte extracellular matrix, and knockdown of circRNAs‐MSR could be a potential therapeutic target for osteoarthritis.^48^ In a recent study, a mitochondrial fission circRNA (MFACR) was shown to mediate cardiomyocyte apoptosis by targeting the miR‐652‐3p‐MTP18 signaling axis, and consequently, the mitochondrial fission resulting in promoting the progress of myocardial infarction.^49^

miRNAs are a general class of endogenous noncoding RNAs of 22–25 nucleotides, widely existing in diverse species, and playing essential roles in cell proliferation, immune response, and maintaining homeostasis.^50^, ^51^, ^52^ miRNAs can control the expression of nearly 30% of protein‐coding genes by targeting a sequence located in the 3′‐untranslated region (3′‐UTR) of the target genes, resulting in cleavage or inhibition of translation and causing profound changes in protein levels.^53^, ^54^, ^55^ Moreover, a single miRNA can simultaneously regulate multiple target genes within a genetic network, resulting in potent cumulative effects on gene networks and affect many biological processes and diseases. The miRNAs formation involved three steps; the first step is when the miRNAs DNA loci transcribed by RNA polymerase II into long primary miRNA transcripts (pri‐miRNAs). Then, the pri‐miRNAs are cleaved into ~100 nt long miRNA precursors pre‐miRNAs by RNase III enzyme, Drosha which is located in the nucleus. Finally, Pre‐miRNAs are subsequently translocated into the cytoplasm via Exportin‐5, where another RNase III enzyme Dicer cleaves off the loop of the pre‐miRNAs and generates mature ~19–25 nucleotide long miRNA duplexes, which may be generated from the 5′ and 3′ arms of the precursor duplex, and are called miRNA‐5p and ‐3p, respectively.^56^, ^57^, ^58^

The first miRNA, lin‐4, was reported in 1993 in *Caenorhabditis elegans*. Lee et al. found that Lin‐4 was negatively regulated the mRNA of the protein‐coding gene lin‐14, which acts as a critical regulator for larval stage development—ensuring the proper transitions between *Caenorhabditis elegans* larval stages. With subsequent studies, every cell type in humans expresses miRNAs, and thousands of miRNAs with potent gene regulatory functions were discovered in almost all organisms.^59^, ^60^ miRNAs can be classified into two broad categories according to their genomic region: intragenic and intergenic. Intragenic miRNAs are located within intronic miRNAs or exonic miRNAs of the protein‐coding genes, whereas intergenic miRNAs are located in the regions between annotated genes. Intergenic miRNAs have their promoters and transcription start site, are expressed independently, and can be regulated by separate transcription factors. At the transcriptional level, expression of miRNA genes can change independently of intergenic miRNAs or together with intragenic miRNAs. On the post‐transcriptional level, the expression of microRNAs can be downregulated due to changes in the activity of key miRNA biogenesis enzymes, such as Drosha and Dicer. Exogenous origin (xenobiotics) and chemical compounds of endogenous origin (hormones) can alter microRNA expression. Moreover, the expression of microRNA also can be regulated by methylation of the promoter.^61^, ^62^, ^63^, ^64^ Analysis of the miRNA target sites indicated that genes with shorter 3′‐UTR usually have a lower density of miRNA binding sites and tend to be involved in basic cellular processes, whereas genes with longer 3′‐UTR usually have a higher density of miRNA‐binding sites and are primarily engaged in developmental regulations.

Three mechanisms have been described, which result in reduced binding of miRNAs to their targets. First, RNA‐binding proteins can inhibit the access of miRNAs to their target sites. Second, the 3′‐UTR length of target genes varies depending on the cell cycle and embryonic development. In proliferating cells, genes with shorter 3′‐UTR can omit their miRNA target sites, resulting in higher protein expression. Finally, miRNAs can be sequestered by mRNAs, lncRNAs, and circRNAs that contain miRNA binding sites and thus acting as a sponge.^65^, ^66^, ^67^, ^68^ Argonaute (Ago) protein is an RNA‐binding protein, which exists in the extracellular space as a free protein or in secreted exosomes along with other RNA‐processing proteins. Ago accumulates in cytoplasmic processing bodies (P‐bodies), where additional binding interactions promote mRNA decay and translational inhibition.^69^, ^70^, ^71^ A ribonucleoprotein complex called the RNA induced silencing complex (RISC), which is formed by Ago proteins, such as Ago‐2, and is involved in target recognition by small noncoding RNAs. Ago‐2 protein separates mature miR duplexes into two strands, collectively known as the guide strand (miR) and the passenger strand (miR*). Then, the factor facilitates incorporation of the miR into RISC complex and guides the RISC miRNA assembly to target mRNAs, whereas the miR* undergoes degradation.^72^, ^73^, ^74^ The miRNA guides RISC to mRNAs that have miRNA complementary sites and RISC then cleaves, degrades, or suppresses translation of the target mRNA, depending on the degree of complementarity between “miRNA response elements” on target mRNA sequences and mature miRNA seed sequences (usually at the second through ninth nt in the core region of the miRNA). In the case of perfect complementarity, the Ago‐containing RISC engages deadenylation enzymes/decapping to cause the target mRNA undergoes a degradation process. Conversely, partial complementarity may block the translation machinery.^66^, ^75^, ^76^

As an important member of noncoding RNA, microRNAs have been demonstrated to participate in each phase of autophagy. For instance, c‐MYC is one of the essential transcriptional factors, regulating a diverse array of cellular functions, including proliferation, growth, and apoptosis. Lu et al. provide evidence showing that increased c‐Myc in Crohn's disease individuals enhances miR106B and miR93 expression, which reduces autophagosome formation and intracellular bacterial removal by targeting ATG16L1.^77^ The phase of vesicle initiation was suppressed by miR‐376b, miR‐17‐5p, miR‐216a, and miR‐30a/b inhibiting BECLIN1 expression. Elongation stage was inhibited by miR‐204 that directly targets LC3. By miR‐101, miR‐34a, miR‐24‐3p, and miR‐376b that modulates ATG4.^78^ The upregulation of miR‐423‐5p could inhibit autophagosome maturation through suppressing autophagosome–lysosome fusion in macrophages.^79^ Next, we will further elaborate on the regulatory role of microRNAs in autophagy in different disease phenotypes.

### 1.1 miRs regulate autophagy in cancer

miRs function as tumor suppressor genes in most cancers to regulate tumor cells' various biological processes, including inhibiting cancer cell metabolism, proliferation, invasion, and migration and inducing cell autophagy and apoptosis. A growing number of studies have confirmed that miRNAs play essential roles in the diagnosis, development, prognosis, and treatment of a variety of tumors. miRs will become potential therapeutic targets, anticancer agents, and biomarkers for the treatment of cancer and bring novel guidance in molecular targeting treatment of cancers in the future.^80^, ^81^, ^82^, ^83^ Approximately about 85% of lung diseases are associated with non‐small cell lung cancer (NSCLC), it is one of the most leading causes of cancer mortality and the most prevalent cancers. Its development and occurrence are closely associated with tumor angiogenesis.^84^, ^85^, ^86^ A transforming growth factor (TGF‐β) is a multifunctional cytokine. c‐Jun N‐terminal kinase (JNK), also known as a stress‐activated protein kinase (SAPK) of the MAPK family, is initially activated in response to a variety of stress signals. miR‐26 inhibited cell autophagy of NSCLC, through inhibiting TGF‐β expression in a JNK‐dependent manner, both in vitro and in vivo.^87^ Collagen α‐1(X) chain (COL10A1), which encodes the α chain of type X collagen, is confirmed to be a member of the collagen family. Outcomes in vivo and in vitro suggested that miR‐384 downregulated COL10A1 by targeting it, subsequently inhibiting cell proliferation and promoting cell autophagy in NSCLC cells.^88^

Gastric cancer is one of the most lethal cancers in the digestive system, with high morbidity and mortality rates worldwide.^89^, ^90^ miR‐1265 suppresses GC progression and oncogenic autophagy by downregulating calcium‐binding protein 39 (CAB39) expression and regulating the AMPK mTOR signaling pathway.^91^ snoRNA host gene 5 (SNHG5) promoted GC cell apoptosis and autophagy by suppressing the expression of miR‐20a.^92^ Cervical cancer is the second most prevalent cancer type in women worldwide, carrying high risks of mortality and morbidity. circRNA hsa_circ_0023404 was significantly upregulated in CC tissues compared to adjacent normal tissues. And, it enhances cervical cancer metastasis and chemoresistance through vascular endothelial growth factor A (VEGFA) and autophagy signaling by sponging miR‐5047.^93^ GRSF1 (G‐rich RNA sequence binding factor 1) was originally identified as an RNA‐binding protein with high affinity for G‐rich sequences. MIR‐G‐1 upregulates TMED5 and lamin B1 (LMNB1) in a GRSF1‐dependent manner and promotes malignant behavior and nuclear autophagy in the cervical cancer cells.^94^

Ovarian cancer is one of the prevalent cancers in perimenopausal women. The notch is highly expressed in the neural precursor cells and plays a critical role in regulating neural differentiation and neural proliferation.^95^, ^96^ Overexpression of miR‐34 inhibits the proliferation of ovarian cancer cells by inducing apoptosis and autophagy via targeting of Notch receptor 1 (Notch 1).^97^ AlkB homolog 5 (ALKBH5) is an N6‐methyladenosine (m6A) eraser protein. And, it inhibited autophagy of epithelial ovarian cancer through miR‐7 and regulated ovarian cancer proliferation and invasion via B cell lymphoma 2 (Bcl‐2).^98^ MiR‐338‐5p promotes metastasis of colorectal cancer by inhibition of phosphatidylinositol 3‐kinase, catalytic subunit type 3‐mediated autophagy pathway.^99^ Pancreas ductal adenocarcinoma (PDAC) is one of the most common gastrointestinal malignancies. The high‐expression level of small nucleolar RNA host gene 14 (SNHG14) could significantly promote proliferation, migration, and invasion abilities of PDAC cells. SNHG14 enhances gemcitabine resistance by sponging miR‐101 to stimulate cell autophagy in pancreatic cancer.^100^ MicroRNA‐137 inhibits autophagy and chemo sensitizes pancreatic cancer cells by targeting ATG5.^101^ Osteosarcoma (OS) is the most common malignant bone tumor. Sphingosine kinase 1 (SPHK1), as a cytosolic enzyme, maintains the intracellular sphingolipid balance and plays vital roles in cell growth, invasion, and autophagy. MicroRNA‐506‐3p initiates mesenchymal‐to‐epithelial transition and suppresses autophagy in osteosarcoma cells by directly targeting SPHK1 (Table 1).^102^

**Table 1:** miRs regulate autophagy in cancer

miRNAs	miRNA status in cancer	Effect on autophagy	Target	Disease	Reference
miR‐26	Downregulated	Inhibition	TGF‐β1‐JNK	NSCLC	87
miR‐384	Downregulated	Activation	COL10A1	NSCLC	88
miR‐1265	Upregulated	Inhibition	AMPK‐mTOR	GC	91
miR‐20a	Downregulated	Activation	SNHG5	GC	92
miR‐5047	Downregulated	Inhibition	VEGFA	Cervical cancer	93
miR‐G‐1	Upregulated	Activation	TMED5 and LMNB1	Cervical cancer	94
miR‐34	Downregulated	Activation	Notch 1	Ovarian cancer	97
miR‐7	Downregulated	Activation	BCL‐2	Ovarian cancer	98
miR‐338‐5p	Upregulated	Inhibition	PIK3C3	CRC	99
miR‐101	Downregulated	Activation	SNHG14	PDAC	100
miR‐137	Upregulated	Inhibition	Dox	PC	101
miR‐506‐3p	Downregulated	Inhibition	SPHK1	OS	102

### 1.2 miRs regulate autophagy in atherosclerosis

Atherosclerosis (AS) underlies most cardiovascular diseases, is characterized by the accumulation of lipids and the proliferation of fibrous materials, proliferation of arterial smooth muscle cells, and chronic inflammatory cells.^103^, ^104^, ^105^ miR‐33 has potent effects on autophagy and lysosomal function that reinforce its targeting of cholesterol efflux and reverse cholesterol transport gene pathways to mediate cholesterol homeostasis. miR‐33 inhibition in atherosclerotic mice restores defective autophagy in the aorta and macrophages of atherosclerotic plaques.^106^ miR‐155 plays a crucial role in AS development, including promoting autophagosome and autolysosome accumulation. It has a promoting effect on oxidized low‐density lipoprotein (ox‐LDL)‐induced autophagy in human umbilical vein endothelial cells (HUVECs).^107^ Endothelial cell injury and subsequent death play an essential role in the pathogenesis of AS. Upregulation of miR‐30 by high‐fat diet (HFD) may impair the protective effects of endothelial cell autophagy against the development of AS through suppressing protein translation of Atg6.^108^ Upregulation of miR‐129‐5p by HFD may impair the protective effects of endothelial cell autophagy against the development of AS through suppressing protein translation of Beclin‐1.^109^ Macrophage autophagy regulated by miR‐384‐5p‐mediated control of Beclin‐1 plays a role in the development of AS. The decreases in Beclin‐1 in macrophages were due to HFD induced increases in miR‐384‐5p, which suppressed the translation of Beclin‐1 mRNA via 3′‐UTR binding.^110^

miR‐126 is a crucial regulator of AS. miR‐126 alleviates ox‐LDL‐induced HUVECs injury through restoring autophagy flux via repressing PI3K/Akt/mTOR pathway and further implicates the potential therapeutic targets to reverse AS.^111^ miR‐214‐3p regulates ox‐LDL‐initiated autophagy in HUVECs by directly targeting the 3′‐UTR of ATG5 and may have a suitable role in the pathogenesis of AS.^112^ The development and function of macrophages are shaped by micro‐environmental signals, which drive macrophage differentiation, with the M1 (promote inflammation) and M2 (inhibit inflammation) populations being the two extreme phenotypes of the macrophage polarization spectrum. Threose nucleic acid (TNA) is refractory to nuclease digestion and capable of undergoing Darwinian evolution to produce aptamers with affinity to specific targets. And, it could activate KLF4 and enhance autophagy as well as M2 polarization of macrophages by inhibiting miR‐375 to attenuate AS.^113^ miR‐21 restored impaired autophagic flux and lysosomal dysfunction, thereby attenuating ox‐LDL‐induced human aortic endothelial cells (HAECs) injuries in AS (Table 2).^114^

**Table 2:** miRs regulate autophagy in atherosclerosis

miRNAs	miRNA status in atherosclerosis	Effect on autophagy	Target	Disease	Reference
miR‐33	Downregulated	Activation	Atg5	AS	106
miR‐155	Upregulated	Activation	Ox‐LDL	AS	107
miR‐30	Upregulated	Inhibition	ATG6	AS	108
miR‐129‐5p	Upregulated	Inhibition	Beclin‐1	AS	109
miR‐384‐5p	Upregulated	Inhibition	Beclin‐1	AS	110
miR‐126	Upregulated	Activation	Ox‐LDL	AS	111
miR‐214‐3p	Upregulated	Inhibition	ATG5	AS	112
miR‐375	Upregulated	Activation	KLF4	AS	113
miR‐21	Downregulated	Activation	Ox‐LDL	AS	114

### 1.3 miRs regulate autophagy in cardiac disease

Pathological cardiac hypertrophy (CH) is a primary risk factor for almost all forms of heart failure and is a common pathological change frequently accompanied by chronic hypertension, disruption of sarcomeric structure, and myocardial infarction. miR‐365 modulates autophagy in CH by decreasing S‐phase kinase‐associated protein 2 (SKP2) and mTORC1 signaling under the induction of CH.^115^ Transient receptor potential vanniloid 3 (TRPV3) was a direct target of miR‐103. miR‐103 could attenuate cardiomyocyte hypertrophy partly by reducing cardiac autophagy activity through the targeted inhibition of TRPV3 signaling in the pressure‐overloaded rat hearts.^116^ miR‐199a impairs autophagy and induces CH through mTOR activation.^117^ Tuberous sclerosis complex 1 (TSC1) was a direct target of miR‐451. miR‐451 is one of the most downregulated microRNAs in hypertrophic cardiomyopathy (HCM) and regulates CH and cardiac autophagy by targeting TSC1.^118^

cardiac‐specific overexpression of miR‐222 could induce pathological cardiac remodeling and heart failure. miR‐221‐induced cardiac remodeling is associated with the downregulation of p27 kip1 (p27), activation of the mTOR pathway, and the subsequent inhibition of autophagy in cardiomyocytes.^119^ Cardiac fibrosis is characterized by the net accumulation of extracellular matrix proteins in the cardiac interstitium and contributes to compromised cardiac function and potentially heart failure. TGF‐β1 plays an essential role in fibrogenesis in heart disease. Autophagy inhibition of hsa miR‐19a‐3p/19b‐3p by targeting TGF‐β R II during TGF‐β1‐induced fibrogenesis in human cardiac fibroblasts.^120^ ULK1 is a critical component in the autophagy pathway. MiR‐26a‐5p regulates cardiac fibroblasts collagen expression by targeting ULK1 (Table 3).^121^

**Table 3:** miRs regulate autophagy in cardiac disease

miRNAs	miRNA status in cardiac disease	Effect on autophagy	Target	Disease	Reference
miR‐365	Upregulated	Inhibition	Skp2	CH	115
miR‐103	Upregulated	Inhibition	TRPV3	CH	116
miR‐451	Downregulated	Activation	TSC1	CH	118
miR‐221	Upregulated	Inhibition	p27	Heart failure	119
miR‐19a‐3p/19b‐3p	Downregulated	Activation	TGF‐β1	Cardiac fibrosis	120
miR‐26a‐5p	Upregulated	Activation	ULK1	Cardiac fibrosis	121

### 1.4 miRs regulate autophagy in tuberculosis

Worldwide, tuberculosis (TB) is an infectious bacterial disease that is one of the top 10 causes of death, and millions of people continue to fall sick with it each year. *Mycobacterium tuberculosis* (MTB) is an intracellular pathogen that causes tuberculosis. It possesses a complex cell wall with a thin peptidoglycan layer that acts as a protective barrier on the cell membrane and a scaffold for the attachment of proteins and polymers. Survival mechanisms of MTB are the inhibition of phagosomes acidification and maturation. It is thereby rendering the intraphagosomal environment more compatible with bacterial survival and replication.^122^, ^123^, ^124^

miR‐30A, an autophagy‐related microRNA, acts as a negative regulator of autophagy in MTB infected macrophages and that H37Rv triggered an increase in miR‐30A levels may play an important role in mediating the escape of MTB from killing by macrophages due to inhibited autophagic pathways.^125^ DRAM2 (DNA damage‐regulated autophagy modulator 2) is a crucial coordinator of autophagy activation that enhances antimicrobial activity against MTB. MIR144 plays a role in the inhibition of autophagy induction and autophagic flux. Overexpression of MIR144 decreased DRAM2 expression and formation of autophagosomes in human monocytes, suggesting the clinical significance of MIR144‐DRAM2 in TB infection.^126^ KLF4 tilts the macrophage response toward the production of arginase and inhibition of autophagy. miR‐26a/KLF4 and CREB‐C/EBPβ signaling pathways play crucial roles in regulating the survival of MTB in macrophages, including regulating innate immune signaling, the polarization of macrophages, and the trafficking of *Mycobacterium tuberculosis* to lysosomes during infection.^127^ Upregulation of miR‐129‐3p decreased mRNA or protein level of Atg4b in RAW264.7 cells and resulted in the inhibition of autophagy and favored intracellular BCG survival.^128^ Calcium channel, voltage‐dependent, alpha‐2/delta subunit 3 (CACNA2D3) is an auxiliary member of the alpha‐2/delta subunit family of the voltage‐dependent calcium channel complex. Induction of miR‐27a was found to target the Ca2 transporter Cacna2d3 directly and downregulate ER Ca2 signaling to inhibit autophagy, thus promoting the intracellular survival of MTB.^129^

lncRNA, PCED1B‐AS1, modulates macrophage apoptosis and autophagy by targeting the miR‐155 axis inactive TB. It may represent a novel early diagnostic marker of active TB and may be useful in the development of potential therapeutic interventions for patients with TB.^130^ miR‐106b‐5p is a potential target for host‐directed therapy for MTB infection. And, it indeed targets the 3′‐untranslated region of cathepsin S (CtsS) mRNA to control CtsS expression resulting in higher pathogen survival and poor T‐cell activation by interfering with proteolysis in the endolysosomal pathway and independently of autophagy and programmed cell death activation.^131^ MTB survives within macrophages by evading delivery to the lysosome and promoting the accumulation of lipid bodies, which serve as a bacterial source of nutrients. miR‐33 and miR‐33* regulate autophagy through both direct targeting of autophagy effectors and by repressing AMPK‐dependent activation of autophagy and lysosomal gene transcription, and these regulatory mechanisms are engaged in MTB infection.^132^ miR‐155 negatively regulates ATG3, an E2‐ubiquitin‐like conjugating enzyme involved in autophagosome formation, thereby impairing autophagy and favoring intracellular MTB survival (Table 4).^133^

**Table 4:** miRs regulate autophagy in tuberculosis

miRNAs	miRNA status in tuberculosis	Effect on autophagy	Target	Disease	Reference
miR‐30A	Upregulated	Inhibition	Beclin‐1	Tuberculosis	125
miR‐144	Upregulated	Inhibition	DRAM2	Tuberculosis	126
miR‐26a	Upregulated	Inhibition	KLF4	Tuberculosis	127
miR‐129‐3p	Upregulated	Inhibition	Atg4b	Tuberculosis	128
miR‐27a	Upregulated	Inhibition	Cacna2d3	Tuberculosis	129
miR‐155	Upregulated	Activation	PCED1B‐AS1	Tuberculosis	130
miR‐106b‐5p	Upregulated	Activation	CtsS	Tuberculosis	131
miR‐33/miR‐33	Upregulated	Inhibition	ATG	Tuberculosis	132
miR‐155	Upregulated	Activation	Atg3	Tuberculosis	133

### 1.5 miRs regulate autophagy in viral diseases

Dengue virus (DENV) is a positive single‐stranded RNA virus of the family Flaviviridae, and dengue is a mosquito‐borne viral disease of increasing incidence and expanding the geographical range.^134^, ^135^ Tumor necrosis factor receptor‐associated factor 6 (TRAF6) promotes autophagy by supporting ULK1 ubiquitination. miR 146a inhibits DENV‐induced autophagy by targeting TRAF6.^136^ Hepatocellular carcinoma is one of the deadliest types of cancer and is one of the highest overall mortality compared to others. Hepatitis B virus (HBV) is a significant risk factor for the development and progression of hepatocellular carcinoma. miRNAs can modulate host gene expression and thereby inhibit or enhance HBV replication.^137^, ^138^, ^139^ The miR‐99 family promotes HBV replication post‐transcriptionally through insulin‐like growth factor 1 receptor (IGF‐1R)/phosphoinositide 3‐kinase (PI3K)/protein kinase B (Akt)/mTOR/ULK1 signaling‐induced autophagy.^140^ miR‐192‐3p is a regulator of HBV infection and may play a potential role in hepatocellular carcinoma. It may also serve as a biomarker or therapeutic target for HBV patients. HBV induces autophagy through the axis of miR‐192‐3p‐XIAP (X‐linked inhibitor of apoptosis [XIAP]) through NF‐kappa B signaling, and that autophagy may be essential for HBV replication.^141^ p53 is one of the most intensively studied tumor suppressor proteins. miR‐146a‐5p promoted HBV replication through the XIAP‐mediated MDM2 (mouse double minute 2 [MDM2])/p53 autophagy pathway to promote aggravation of chronic hepatitis B.^142^

Enterovirus 71 (EV71) is a group of viruses that belongs to the Picornaviridae family and is the principal causative agent of severe and fatal hand, foot, and mouth disease. Overexpression of miR‐30a could suppress EV71 replication by blocking EV71‐induced autophagy.^143^ The 14‐3‐3 proteins are among the most abundant proteins expressed in the brain, comprising about 1% of the total amount of soluble brain proteins. Through phosphoserine‐ and phosphothreonine‐binding motifs, 14‐3‐3 proteins regulate many signaling proteins and cellular processes, including cell death. Porcine circovirus type 2 (PCV2) is the primary causative agent of porcine circovirus‐associated disease (PCVAD), and although small, it has the highest evolution rate among DNA viruses. Overexpression of miR‐30a‐5p triggered PCV2‐induced autophagosomes formation and facilitated PCV2 replication in a dose‐dependent manner by targeting 14‐3‐3.^144^ Rotavirus (RV), the principal etiological agent of viral gastroenteritis in young children, kills over 200 thousand infants each year. miR‐99b modulates the expression of mTOR following RV infection and modulates autophagy for successful RV replication.^145^

Molecular assays for detection and accurate quantitation of hepatitis C virus (HCV) RNA have been important for identification and management of the hepatitis C. ATG5, a target gene for miR‐130a, significantly upregulated HCV replication and downregulated interferon‐stimulated gene expression. And, miR‐130a regulates host antiviral response and HCV replication through targeting ATG5 via the ATG5‐dependent autophagy pathway.^146^ Bovine viral diarrhea virus (BVDV), the causative agent of bovine viral diarrhea/mucosal disease (BVD/MD), is an important pathogen associated with reproductive, gastrointestinal, and respiratory diseases. Lentivirus‐mediated bta‐miR‐29b overexpression interferes with BVDV replication and viral infection‐related autophagy by directly targeting ATG14 and ATG9A in Madin‐Darby bovine kidney (MDBK) cells (Table 5).^147^

**Table 5:** miRs regulate autophagy in viral diseases

miRNAs	miRNA status in viral diseases	Effect on autophagy	Target	Disease	Reference
miR‐146a	Upregulated	Activation	TRAF6	Dengue	136
miR‐99	Upregulated	Inhibition	IGF 1R	Hepatocellular carcinoma	140
miR‐192‐3p	Downregulated	Inhibition	NF kappa B	Hepatocellular carcinoma	141
miR‐146a‐5p	Upregulated	Inhibition	MDM2	Chronic hepatitis B	142
miR‐30a	Downregulated	Activation	Beclin‐1	Hand‐foot‐and‐mouth disease	143
miR‐30a‐5p	Upregulated	Activation	BCL2L2	PCVAD	144
miR‐99b	Upregulated	Inhibition	Let‐7g	Viral gastroenteritis	145
miR‐130a	Upregulated	Inhibition	ATG5	Hepatitis C	146
miR‐29b	Upregulated	Inhibition	ATG14/ATG9A	BVD/MD	147

## 2 PERSPECTIVES

In this review, we illustrate that microRNAs play an important regulatory role in autophagy through several examples and may be able to provide new ideas for studying the possible role of miRs on regulating the press of various diseases. MicroRNA may act as a regulator of many diseases to influence disease processes and biological processes, including inhibition of cell proliferation, migration and invasion, and induction of autophagy and apoptosis. Understanding the regulation mechanism of microRNA on autophagy will provide a new way to further study its functional activity and microRNA‐based therapy for patients with new diseases. Moreover, for the expression of microRNAs under different conditions, autophagy regulation and the selection of effective target genes for cross‐disease treatment show more research significance and reference value. More and more microRNAs are regarded as potential targets for disease control and treatment. The potential value of microRNAs used in clinical practice has been generally accepted, and microRNAs have become a potential factor for early diagnosis of many diseases and vaccine development of related diseases. Deeply revealing the function of host microRNAs in regulating autophagy may help to develop microRNAs‐based approaches to host‐directed therapy for infections, inflammation, and pathogen killing.

## CONFLICTS OF INTEREST

The authors declare that there are no conflicts of interest in this manuscript.
